# Comparative Genome-Wide Identification of the *Fatty Acid Desaturase* Gene Family in Tea and Oil Tea

**DOI:** 10.3390/plants13111444

**Published:** 2024-05-23

**Authors:** Ziqi Ye, Dan Mao, Yujian Wang, Hongda Deng, Xing Liu, Tongyue Zhang, Zhiqiang Han, Xingtan Zhang

**Affiliations:** 1 The Laboratory of Forestry Genetics, Central South University of Forestry and Technology, Changsha 410004, China; 20211100001@csuft.edu.cn (Z.Y.); 20211200002@csuft.edu.cn (H.D.); 20221100002@csuft.edu.cn (X.L.); 20221100001@csuft.edu.cn (T.Z.); 2 National Forest and Seedling Workstation of Hunan Province, The Forestry Department of Hunan Province, Changsha 410004, China; ludovico2024@163.com (D.M.); 19155506489@163.com (Y.W.); 3 Agricultural Genomics Institute at Shenzhen, Chinese Academy of Agricultural Sciences, Shenzhen 518000, China

**Keywords:** fatty acid desaturase gene family, phylogenetic analysis, gene expression, fatty acid biosynthesis

## Abstract

Camellia oil is valuable as an edible oil and serves as a base material for a range of high-value products. *Camellia* plants of significant economic importance, such as *Camellia sinensis* and *Camellia oleifera*, have been classified into sect. *Thea* and sect. *Oleifera*, respectively. Fatty acid desaturases play a crucial role in catalyzing the formation of double bonds at specific positions of fatty acid chains, leading to the production of unsaturated fatty acids and contributing to lipid synthesis. Comparative genomics results have revealed that expanded gene families in oil tea are enriched in functions related to lipid, fatty acid, and seed processes. To explore the function of the *FAD* gene family, a total of 82 *FAD* genes were identified in tea and oil tea. Transcriptome data showed the differential expression of the *FAD* gene family in mature seeds of tea tree and oil tea tree. Furthermore, the structural analysis and clustering of FAD proteins provided insights for the further exploration of the function of the *FAD* gene family and its role in lipid synthesis. Overall, these findings shed light on the role of the *FAD* gene family in *Camellia* plants and their involvement in lipid metabolism, as well as provide a reference for understanding their function in oil synthesis.

## 1. Introduction

The genus *Camellia*, a member of the family Theaceae, is one of the largest and most economically valuable genera in the family [1]. It includes many representative species, such as *Camellia sinensis*, *Camellia oleifera*, and *Camellia chekiangoleosa*, which have significant applications in tea leaves and oil production, as well as ornamental purposes [2,3,4]. These plants exhibit variations in plant morphology, economic properties, and oil content composition [5,6]. The basic metabolic pathways of fatty acid biosynthesis have been extensively studied in model plants, with a particular focus on important oil-producing plants, such as olive, soybean, and peanut, that regulate the accumulation of oleic acid and linoleic acid [7,8,9]. However, there is an urgent need for functional genomics research on fatty acid biosynthesis pathways specific to oil tea plants. In recent years, important progress has been achieved in the assembly and annotation of oil tea genomes, providing a foundation for exploring potential genetic markers and genes, and promoting the transition from traditional breeding to genomics-assisted breeding in oil tea production. Among them, *C. oleifera* var. “Nanyongensis” (CON), recognized as the wild progenitor of cultivated diploid oil tea, holds considerable significance [10]. Meanwhile, *C. chekiangoleosa*, a prominent oil tea plant, has a long tradition of cultivation in southern China [11]. Within the section *Oleifera*, *C. lanceoleosa*, a wild diploid species, has primarily evolved through natural selection, endowing it with distinctive characteristics [12]. Decoding the genomes of these oil tea plants could help identify potential genetic markers and genes, enabling a transition from traditional breeding to genomics-assisted breeding in oil tea production.

Fatty acid desaturases (FADs) play a critical role in plant lipid metabolism by introducing double bonds into hydrocarbon chains, thus, influencing the composition of fatty acids in oils [13]. Numerous *FAD* genes have been isolated and characterized from various plants, including soybean, peanut, and tung tree [14,15,16]. In soybean roots, the transcription levels of *GsSLD1* and *GsSLD2* genes are significantly up-regulated after 1 and 2 h of salt stress, indicating that these *FAD* genes may regulate the cell membranes of soybean [17]. In Arabidopsis *FAD6* mutants, polyunsaturated fatty acid levels in chloroplast membranes drop significantly under cold stress, reducing cold tolerance [18]. In bananas, *Fusarium oxysporum f. sp. cubense* tropical race 4 (*Foc*TR4) affects the expression of several *MaFADs* and greatly induces the accumulation of fatty acids in roots [19]. *FAD* genes play pivotal roles in various aspects of plant life, including growth, development, and responses to environmental stresses. *FAD* genes have been found in various plants, including *Triticum aestivum*, *Oryza sativa*, *Musa acuminata*, *Glycine max*, *Brassica napus*, and others [17,19,20,21,22].

Based on their subcellular localization, membrane-bound FADs can be classified into plastidial FADs (FAD4, FAD5, FAD6, FAD7, and FAD8), which desaturate membrane lipids within plastid membranes, and endoplasmic reticulum FADs (FAD2 and FAD3), responsible for synthesizing unsaturated glycerides [23]. Among them, FAD2 in oil tea plays a crucial role in converting oleic acid to linoleic acid and is a key enzyme in oil synthesis and metabolism [24]. In existing studies, there has been extensive research on the seed oil content and unsaturated fatty acid composition in *Camellia* species. Results have shown that the seed oil content in *Camellia* species is generally high, with some species reaching over 40%. An analysis of 25 *Camellia* species in experiments revealed that the majority have an unsaturated fatty acid content exceeding 85%, with 14 species having oleic acid and linoleic acid contents above 85% [25]. Additionally, another study found that *C. oleifera* and *Camellia albogigas*, among other *Camellia* species, have high unsaturated fatty acid proportions, reaching 86% and 83%, respectively, while *C. sinensis* has a comparatively lower unsaturated fatty acid content at 69.60% [26]. These research findings indicate that *Camellia* species seeds are generally rich in unsaturated fatty acids, with oleic acid being a predominant component. Due to artificial selection pressures, oil tea plants have transitioned into being a woody oil crop that is primarily cultivated for the production of seed oil [10]. This domestication process has rendered oil tea an ideal model for studying the genetic and physiological mechanisms underlying the traits associated with seed oil production and domestication. In this study, we conducted a genome-wide identification of *FAD* genes in tea and oil tea, characterized their gene structures, chromosomal distributions, cis-acting elements, conserved motifs, and the phylogenetic relationships of their proteins. Additionally, we performed a comparative analysis of gene expression within the *FAD* gene family in tea and oil tea to uncover the mechanisms of fatty acid accumulation in seeds.

## 2. Results

### 2.1. Genome Evolution and Expansion Analyses

**Figure 1:**
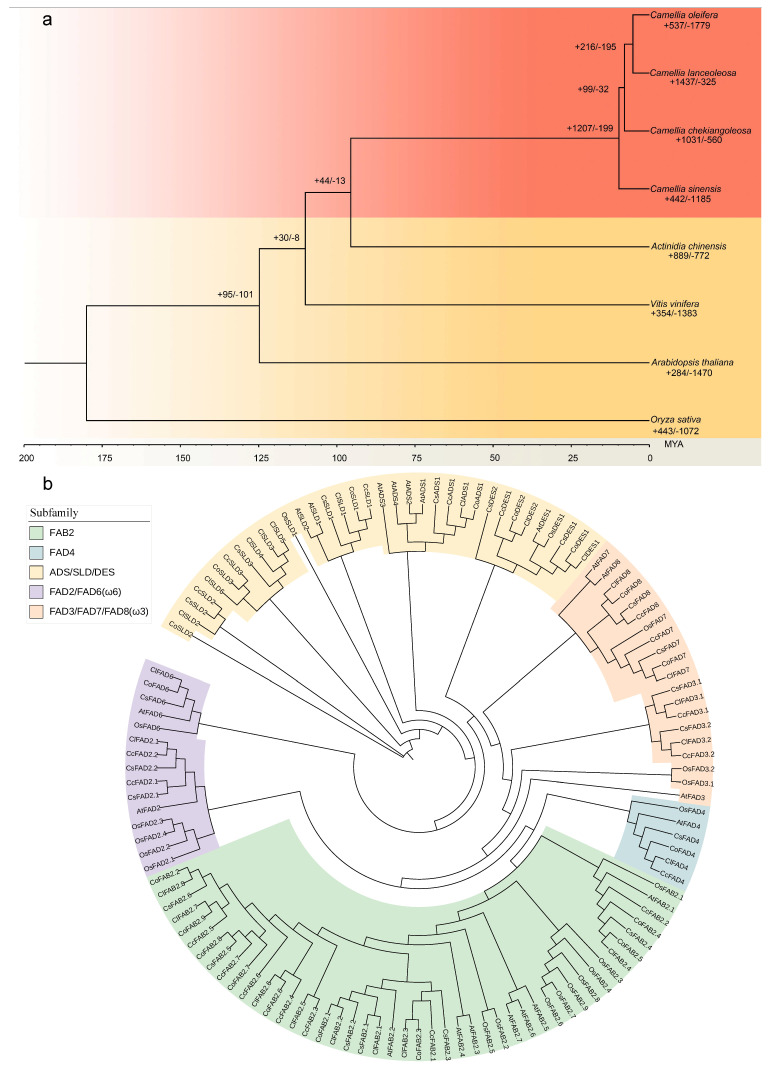
(**a**) Phylogenetic analysis using orthovenn3 with the orthofinder algorithm and the JTT+CAT model. The phylogenetic tree outlines the evolutionary timeline of the species. Species divergence times were determined based on information from the Timetree website. (**b**) Phylogenetic tree depicting the evolutionary relationships among FAD proteins from tea and oil tea (*C. oleifera*, *CoFADs*; *C. chekiangoleosa*, *CcFADs*; *C. lanceoleosa*, *ClFADs*; *C. sinensis*, *CsFADs*; *A. thaliana*, *AtFADs*; and *O. sativa*, *OsFADs*). The tree was constructed using the raxml-ng algorithm, with a total of 1000 bootstrap replications to ensure robustness. Different colors represent distinct subfamilies within the FAD protein family.

A phylogenetic tree was constructed on the basis of 391 single-copy gene families from *C. oleifera*, *C. chekiangoleosa*, *C. lanceoleosa*, *C. sinensis*, *Arabidopsis thaliana*, *Oryza sativa*, *Vitis vinifera*, and *Actinidia chinensis* (Figure 1a). We identified a total of 6923 gene families that were common to all eight species. Additionally, we observed 881 gene clusters that were shared among *C. sinensis*, *C. oleifera*, *C. chekiangoleosa*, and *C. lanceoleosa* (Figure S1). A total of 1437 families, significantly expanded in the *C. lanceoleosa* lineage (*p* < 0.01), underwent gene ontology (GO) annotations related to functions associated with lipid, fatty acid, and seed processes. Studying lipid and fatty acid profiles in seeds is crucial for understanding seed-specific lipid metabolism and essential fatty acid synthesis. And the functionality related to stress responses, such as “response to salt stress” (GO:0009651), contributes to the adaptability of oil tea to its environment. Furthermore, the phylogenetic tree constructed using single-copy genes revealed multiple branches. Two wild species, *C. oleifera* and *C. lanceoleosa*, were closely connected on a single branch of the evolutionary tree. Specifically, the divergence of *C. lanceoleosa* from *C. chekiangoleosa* occurred approximately 7.9 million years ago, followed by the separation of *C. lanceoleosa* from *C. oleifera* approximately 5.2 million years ago (Figure 1a).

### 2.2. Identification and Bioinformatic Analysis of FAD Gene Family

In total, we successfully identified 24 *ClFAD* genes, 19 *CoFAD* genes, 19 *CcFAD* genes, and 20 *CsFAD* genes within the genome. Compared to the other two varieties, the higher quantity of the *FAD* gene family in *C. lanceoleosa* was consistent with the results of the comparative genomics, suggesting that this oil tea may possess greater activity or a richer potential for fatty acid biosynthesis. We conducted an analysis of the encoded protein size, molecular weight, isoelectric points, and subcellular localization of the *FAD* gene members (Table S2). Their encoded protein size ranged from 229 amino acids to 460 amino acids. The theoretical molecular weight of *FADs* varied from 25,371.67 to 52,663.39 Da, with the isoelectric points (pI) ranging from 5.21 to 9.78. With the exception of *CcFAB2.3*, which was located in the mitochondrion, the majority of the *FAB* gene members were predicted to be primarily localized within the chloroplast and cytoplasm. It was observed that *FAD7* and *FAD8* were, respectively, localized within the plasma membrane and the chloroplast.

According to their distinct functions, membrane-bound *FADs* can be further classified into five subfamilies, including FAB2, FAD4, FAD2/FAD6 (Omega-6 desaturases, ω6), FAD3/FAD7/FAD8 (Omega-3 desaturases, ω3), and ADS/SLD/DES. In these four *Camellia* species, each subfamily collectively contained 30, 4, 8, 14, and 26 *FAD* members, respectively (Figure 1b).

*ClFAD6*, *CoFAD6*, and *CsFAD6* exhibited the highest exon count, each containing 10 exons. In the Omega-3 desaturases subfamily, *FAD3* genes typically feature eight to nine exons, along with eight exons in both *FAD7* and *FAD8*. In the ADS cluster, *ClADS1*, *CsADS1*, and *CcADS1* each had five exons, while *CoADS1* had six exons. Members of the DES and SLD subfamilies typically only had one to two exons (Figure S2).

We identified conserved domains, including FA_desaturase, lipid_DES, cyt-b5, and TMEM189_B, through an analysis with the CDD and SMART databases. In most tea and oil tea *FAD* genes, the FAB2 class contained one lengthy FA_desaturase_2 domain. The FAD4 class contained one B domain of TMEM. The DES class featured one C-terminal FA_desaturase domain and one Lipid_DES domain at its N-terminal side. The SLD class contained a C-terminal FA_desaturase domain and an N-terminal cytochrome b5 (Cyt-b5) domain.

**Figure 2:**
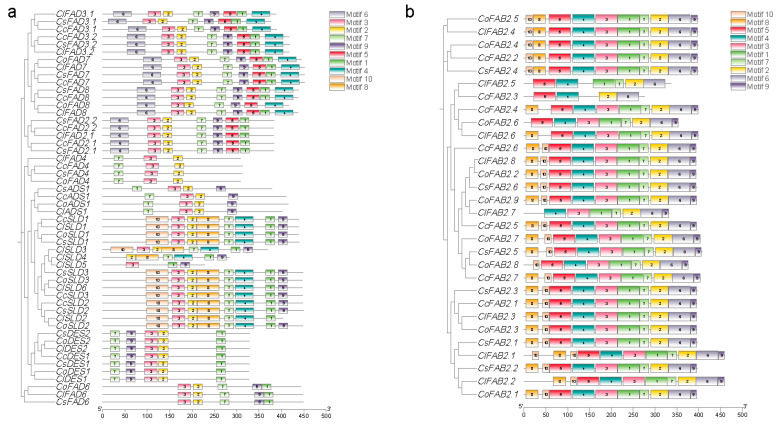
Conservation motif analysis of FAD proteins in tea and oil tea with a set motif search quantity of 10. (**a**) Conserved motifs of FAB2(SAD) subfamily proteins. (**b**) Conserved motifs of FAD proteins, except the FAB2(SAD) subfamily members.

There were 10 motifs identified in the *FAD* gene family (Figure 2). In tea and oil tea, the motif sequence within the FAB2(SAD) subfamily was highly conserved, with the majority following the order of motif 8, 10, 5, 4, 3, 1, 7, 2, 6, 9 (Figure 2b). It could be summarized that FAD proteins within the same subfamily exhibited a substantial similarity in terms of conserved motif types, numbers, and distributions.

**Figure 3:**
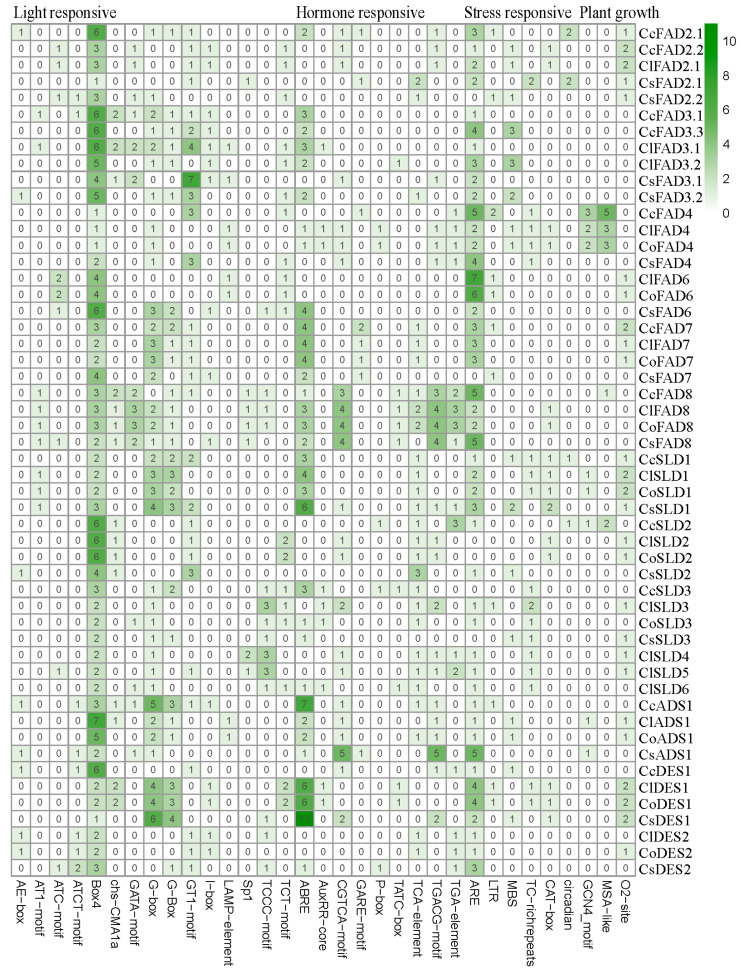
Distribution of cis-acting elements in the promoters of *FAD* gene family, excluding the FAB2(SAD) subfamily members from tea and oil tea.

We conducted a cis-acting elements analysis on the upstream 2 kb sequences of the *FAD* genes using PlantCARE [27] (Figure 3 and Figure S3). To comprehensively investigate the potential biological functions of the *FAD* gene family, we identified 29 distinct elements closely associated with light responsiveness. Light-responsive elements, such as Box 4 (258), were identified in all the promoters of *FADs* except *ClFAB2*.*2*, *CoFAB2*.*1*, and *CsFAB2*.*2*. Notable elements also included G-Box motifs (90), GT1-motif (75), TCT-motif (43), and TCCC-motif (42). Additionally, a spectrum of elements related to plant growth and development emerged, featuring O2-site (45), CAT-box motifs (23), circadian elements (18), and GCN4_motif (15). Remarkably, our analysis unveiled the presence of ARE elements (166), MBS motifs (37), and TC-rich repeats (36), all underscoring their roles as stress-responsive elements. Furthermore, our investigation revealed a multitude of cis-acting elements with hormone responsiveness. This encompassed a substantial number of ABRE motifs (183), indicative of a strong response to abscisic acid. Additionally, we observed TGACG-motif (65), associated with MeJA responsiveness. There were also TCA-element motifs (55), demonstrating a correlation with salicylic acid, alongside P-box sites (22), GARE-motif (17), and TATC-box motifs (16), all indicative of a response to gibberellic acid.

### 2.3. Chromosomal Location and Duplication Events of FAD Genes

**Figure 4:**
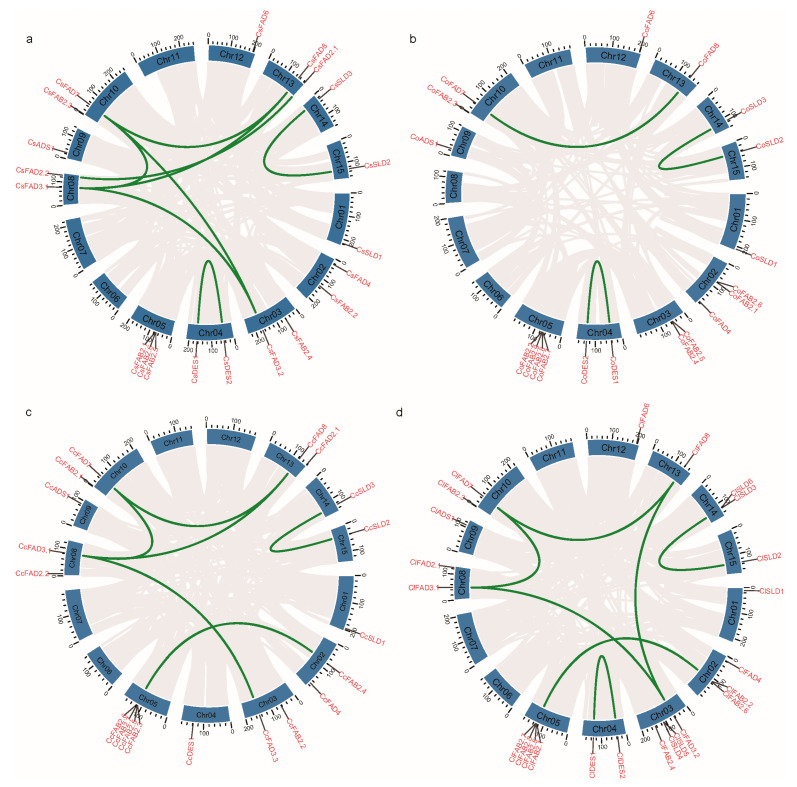
Chromosomal location and collinearity analysis of *FAD* family genes from *C. sinensis* (**a**)*, C. oleifera* (**b**)*, C. chekiangoleosa* (**c**) and *C. lanceoleosa* (**d**). Gene IDs are highlighted in red. Chromosomes are represented by dark blue boxes, and segmental duplication genes are connected with green lines.

In this study, we performed a comprehensive synteny analysis among four tea plant species, namely, *C. sinensis* (Cs), *C. oleifera* (Co), *C. chekiangoleosa* (Cc), and *C. lanceoleosa* (Cl). All four species, each possessing 15 chromosomes, exhibited a robust collinearity (Figure S4). To maintain consistency in describing the chromosomal locations of the gene family members, we adopted the chromosome nomenclature of *C. oleifera*. The chromosomal distribution of genes among the tea plant species (Cs, Co, Cc, and Cl) revealed intriguing patterns (Figure 4). Notably, within the FAD3/FAD7/FAD8 (ω3) subfamily, *FAD7* genes consistently resided on chromosome 10 (Chr10) across all four species, while *FAD8* genes exhibited a commonality in being located on chromosome 13 (Chr13). In the ADS/SLD/DES subfamily, *ADS* genes were clustered on chromosome 9 (Chr09) and *DES* genes were clustered on chromosome 4 (Chr04) for Cs, Co, Cc, and Cl. Furthermore, *SLD* genes demonstrated a shared presence on chromosomes 1 (Chr01), 14 (Chr14), and 15 (Chr15). This shared chromosomal distribution underscored the conserved nature of genomic organization among these tea plant species. Remarkably, the *FAB2* genes on chromosome 5 (Chr05) exhibited closely positioned locations, suggesting a potential origin through duplication events. Specifically, in *C. sinensis* (Cs), the *CsFAB2.1*, *CsFAB2.5*, and *CsFAB2.6* genes were tightly clustered on Chr05 (Figure 4a). In *C. lanceoleosa* (Cl), the *ClFAB2.1*, *ClFAB2.5*, *ClFAB2.7*, and *ClFAB2.8* genes were also located in proximity on Chr05 (Figure 4d). Additionally, the *CoFAB2.2*, *CoFAB2.7*, *CoFAB2.8*, *CoFAB2.9*, *CcFAB2.3*, *CcFAB2.5*, *CcFAB2.6*, and *CcFAB2.7* genes in *C. oleifera* (Co) and *C. chekiangoleosa* (Cc) were tightly clustered on Chr05 as well (Figure 4b,c). This implied a mechanism for the expansion of the *FAD* gene family, indicating that these genes might have arisen through duplication.

To further investigate gene duplication events within the *FAD* gene family, we conducted a collinearity analysis, identifying a total of 24 pairs of segmental duplication genes (Figure 4). Among them, *C. lanceoleosa* displayed seven duplicated gene pairs, including *ClFAD7*/*ClFAD8*, *ClFAD7*/*ClFAD3.1*, *ClFAD3.1*/*ClFAD3.2*, *ClDES1*/*ClDES2*, *ClFAD8*/*ClFAD3.2*, *ClSLD2*/*ClSLD3*, and *ClFAB2.1*/*ClFAB2.2*. *C. sinensis* exhibited eight pairs, such as *CsFAD7*/*CsFAD8*, *CsFAD7*/*CsFAD3.1*, *CsFAD8*/*CsFAD3.1*, *CsFAD3.1*/*CsFAD3.2*, *CsFAD7*/*CsFAD3.2*, *CsDES1*/*CsDES2*, *CsSLD2*/*CsSLD3*, and *CsFAD2.1*/*CsFAD2.2*.

### 2.4. Comparative Expression Analysis of the FAD Gene Family in Seeds of Tea and Oil Tea

**Figure 5:**
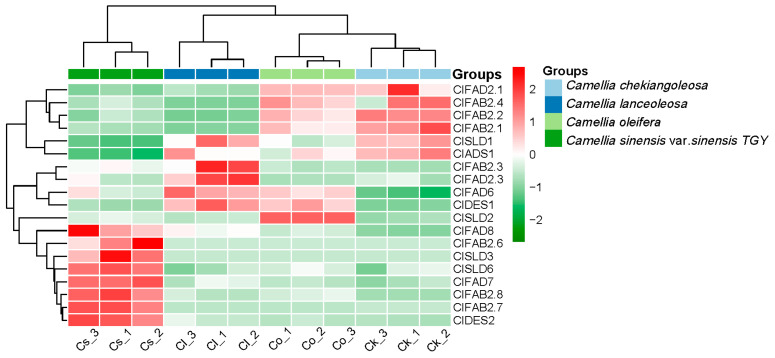
Expression heatmap of *FAD* gene family in tea and oil tea seeds. Among them, the abbreviation “Cl” was used for *C. lanceoleosa*, the abbreviation “Ck” was used for *C. chekiangoleosa*, the abbreviation “Co” was used for *C. oleifera*, and the abbreviation “Cs” was used for *C. sinensis* var. *sinensis TGY*.

Almost all species of the *Camellia* species have seeds from which oil can be extracted. The expression heatmap (Figure 5) revealed that FAD3.2, FAD6, and DES1 were highly expressed in the seeds of *C. lanceoleosa* when comparing the *FAD* gene family between tea and oil tea. Within the FAB2(SAD) subfamily, *FAB2.1*, *FAB2.2*, and *FAB2.4* showed a high expression in *C. oleifera* and *C. chekiangoleosa*. Genes such as *FAD7* and *FAD8* demonstrated relatively high expression levels in *C. sinensis* var. *sinensis TGY*. These findings indicated the differential expression of the *FAD* gene family between tea and oil tea. This may have contributed to the variations in oil contents among seeds of *Camellia* species.

**Figure 6:**
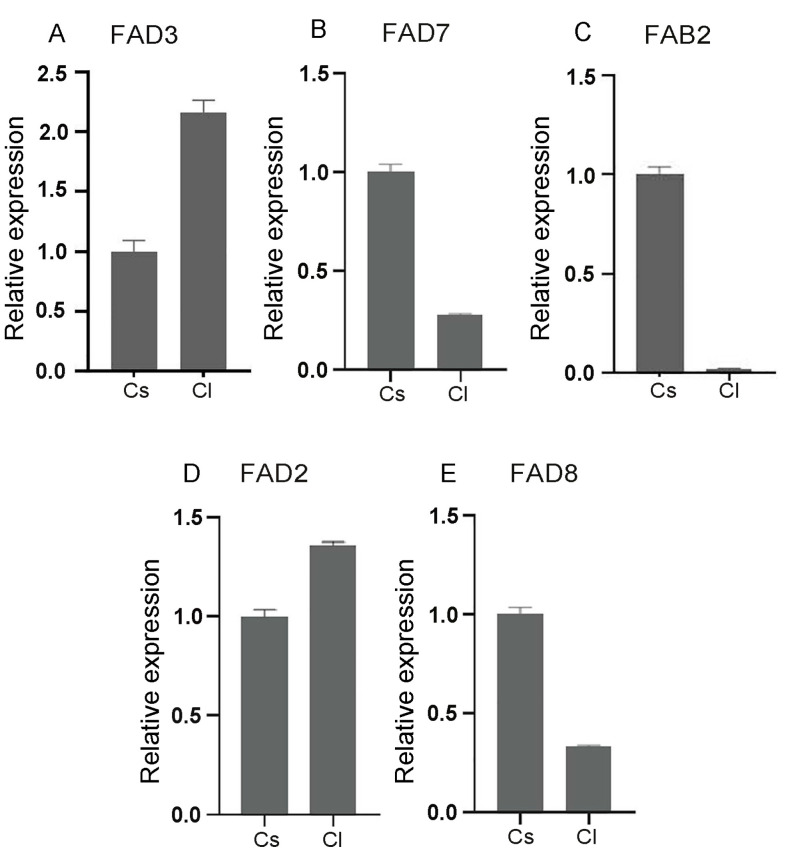
The expression profile of the five representative *FAD* genes using qRT-PCR. Error bars indicate SD (three biological repeats for each sample).

Furthermore, we selected five genes: *FAB2*, *FAD2*, *FAD3*, *FAD7*, and *FAD8*. Their relative expression levels were quantified in the mature seeds of *C. sinensis* var. *sinensis TGY* and *C. lanceoleosa* using qPCR. The analysis indicated that *FAB2*, *FAD3*, *FAD7*, and *FAD8* were significantly differentially expressed in the mature seeds of the tea and oil tea tree (Figure 6).

### 2.5. Clustering and Analysis of FAD Proteins in Tea and Oil Tea

**Figure 7:**
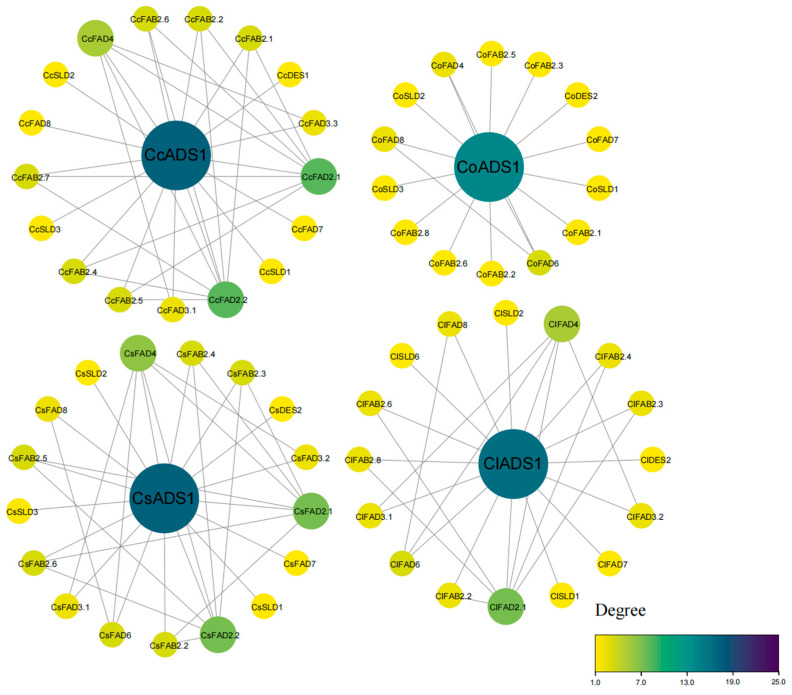
The protein–protein interaction network of the *FAD* gene family in *C. sinensis* (Cs), *C. oleifera* (Co), *C. chekiangoleosa* (Cc), and *C. lanceoleosa* (Cl).

The three-dimensional structure of FAD proteins was primarily composed of α-helices and β-sheets, and some proteins from the same subfamily exhibited similar three-dimensional structures, as exemplified by *CsFAB2*, *ClFAB2*, and *CoFAB2* from the FAB2 subfamily (Figures S5 and S6). The results of the three-dimensional structure predictions suggested that proteins from the same group may share similar functions, indicating potential functional similarities among them. Additionally, we observed that in the FAD protein interaction networks of both tea and oil tea, the aminodeoxychorismate synthase (*ADS*) gene was identified as a hub gene, showing interactions with a majority of *FAD* genes (Figure 7).

**Figure 8:**
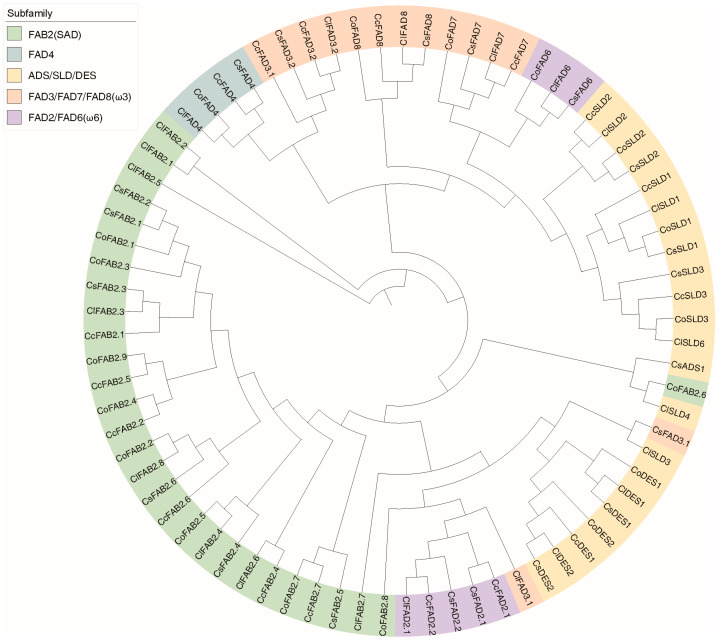
The UPGMA algorithm was used to cluster the FAD proteins based on their protein structure, and the resulting tree was labeled with different colors representing different subfamilies.

We obtained high-confidence protein structures by filtering them based on an average per-residue confidence measure greater than 80, known as the predicted local distance difference test (pLDDT). Five proteins, namely, *CcADS1*, *CcFAB2.3*, *ClADS1*, *ClSLD5*, and *CoADS1*, were filtered out. The remaining 77 FAD proteins were used to construct a tree using the proposed method (Figure 8). The clustering analysis successfully grouped the protein structures based on their structural similarity. The generated UPGMA clustering tree provided a visual representation of the relationships between the structures, revealing distinct clusters and potential evolutionary connections. Further insights into the similarities and differences among the protein structures were gained through pairwise scores, including RMSD and TM-Score.

The differences observed in clustering results between protein structure and sequence could be attributed to the fact that while protein sequences offer insights into the linear arrangement of amino acids, protein structures capture the three-dimensional conformation and spatial arrangement of these amino acids. Protein structures are influenced by various factors, including sequence variations, domain rearrangements, and post-translational modifications [28,29,30]. Even when two proteins share similar sequences, these factors can lead to distinct structural arrangements. Such discrepancies become apparent when comparing clustering outcomes based on structural and sequence similarities. In the case of *CoFAB2.6* and *CsFAD3.1*, their similar predicted protein structures led to them not being grouped into their original subfamilies based on sequence similarity (Figure 8).

## 3. Discussion

We conducted a comparative genomic analysis of tea and oil tea and found that the significantly expanded gene family in oil tea was enriched with functions related to fatty acid synthesis, consistent with previous studies [10,11,12]. After performing a sequence alignment and searching against the Pfam protein family database, we identified 82 candidate *FAD* genes. Among them, the number of *FAD* genes in *C. lanceoleosa* was the largest, indicating that these genes had expanded in evolution and produced many new mutations. The metabolic studies revealed that seeds of *C. lanceoleosa* contained a high oil content of 46.22%, with approximately 80.29% being oleic acid [12]. In contrast, *C. sinensis* seeds had a lower oil content ranging from 26.33% to 31.81% [31], with an oleic acid content of approximately 58% [32]. These results suggested that *C. lanceoleosa* may have higher activity or richer potential for fatty acid biosynthesis. The *FAD* gene family can be divided into five subfamilies, which is similar to the classification observed in banana and wheat [19,22]. And plants have two categories of FAD members: soluble desaturases, like 18:0-ACP desaturase (FAB2/SAD), which function early in fatty acid synthesis, and membrane-bound desaturases, primarily found in the endoplasmic reticulum and chloroplasts [33,34,35]. In *C. sinensis*, *C. lanceoleosa*, *C. oleifera*, and *C. chekiangoleosa*, certain *FAB2* genes were closely located on chromosome 5, forming gene clusters. The chromosome localization analysis suggested a higher level of conservation among members of the same subfamily in oil tea and tea. We found that FAD proteins within the same subfamily shared similarities in terms of the type, number, and distribution of conserved motifs. Similar results have been identified in rice, wheat, and banana, indicating that the *FAD* gene family was conserved [19,20,22]. The difference in conserved motifs among different subfamilies suggested that specific conserved motifs play an important role in plant growth and development.

Our investigation into the protein–protein interaction network of the *FAD* gene family indicated that *ADS* genes likely hold crucial regulatory roles within tea plants and oil tea plants, influencing the function of the *FAD* gene family. Previous research indicated that rice and banana lack *ADS* members, and *ADS* emerged after the divergence of monocotyledonous and dicotyledonous plants [19]. This discovery suggested that *ADS* genes may have distinct evolutionary histories and biological functions across various plant taxa. The functional enrichments in the network included unsaturated fatty acid biosynthesis, the cellular response to cold, and fatty acid biosynthesis as biological processes, along with stearoyl–acyl carrier protein desaturase and acyl–acyl carrier protein desaturase as molecular functions. We identified conserved domains, including FA_desaturase, lipid_DES, cyt-b5, and others. Protein domains are crucial structures that define a protein’s identity and indicate its function [36]. Further, we conducted protein structure predictions for the *FAD* gene family. When considering their structural similarities, these proteins were assigned to different clusters. This could be attributed to the fact that despite having similar sequences, these proteins may have undergone structural changes that resulted in distinct spatial arrangements. Therefore, it is essential to consider both sequence and structural information to gain a comprehensive understanding of protein relationships and evolutionary connections. Previous studies have shown that a structural cluster analysis is more robust than sequence clustering in grouping functionally similar proteins [37]. By incorporating both aspects in the analysis, we could obtain a more comprehensive and accurate depiction of protein clustering and phylogenetic relationships. In existing studies, a novel model has been proposed that integrates protein structure and sequence data, aiming to improve the accuracy of protein function prediction while also enhancing the model’s applicability across different proteins [38]. This study provided a reference for future experiments to verify whether a structural cluster analysis could effectively group proteins with the same function into the same evolutionary branch, potentially using known functional or structurally characterized FAD proteins.

## 4. Materials and Methods

### 4.1. Genome Evolution and Expansion Analyses

OrthoVenn3 [39] (https://orthovenn3.bioinfotoolkits.net/home) was used for the comparative genomics analysis, accessed on 23 September 2023. A phylogenetic tree was constructed on the basis of 391 single-copy gene families from *C. oleifera*, *C. chekiangoleosa*, *C. lanceoleosa*, *A. thaliana*, *O sativa*, *C. sinensis*, *V. vinifera*, and *A. chinensis*. The expansion and contraction of the gene clusters was analyzed with cafe5 [40]. Species divergence times were determined based on information from the Timetree (http://www.timetree.org/, accessed on 23 September 2023).

### 4.2. Identification and Bioinformatic Analysis of FAD Gene Family

The protein sequences for *C. oleifera* var. “Nanyongensis” (CON) can be conveniently accessed on GitHub at the following URL: https://github.com/Hengfu-Yin/CON_genome_data, accessed on 19 July 2023. For *C. sinensis* and *C. chekiangoleosa*, the respective protein sequences are accessible via the Genome Warehouse (GWH) database under the accession numbers GWHASIV00000000 and GWHBGBN00000000. The protein sequences of *O. sativa* and *A. thaliana* were downloaded from the Phytozome database (https://phytozome-next.jgi.doe.gov/, accessed on 19 July 2023).

To identify and characterize members of the *FAD* gene family in oil tea and tea, we utilized a combination of bioinformatics tools. The hidden Markov model (HMM) files corresponding to the FA_desaturase (PF00487), FA_desaturase 2 (PF03405), and TMEM189 (PF10520) domains downloaded from Pfam protein family database (http://pfam.xfam.org/) were searched against the protein data with e-value ≤ 1 × 10^−5^ as the criterion. After removing the redundant sequences, the putative FAD protein sequences were submitted to the NCBI Conserved Domains Database (https://www.ncbi.nlm.nih.gov/cdd/) and SMART database (http://smart.embl.de/), accessed on 28 September 2023.

We constructed phylogenetic trees utilizing RAxML (v1.2.0) [41] with the JTT+FC model based on protein sequences from the *FAD* gene family and performed a bootstrap analysis with 1000 replicates. The MEME (https://meme-suite.org/meme/tools/meme) was utilized with a motif search quantity parameter set to 20 for the purpose of identifying conserved structural domains within the *FAD* gene family, accessed on 15 January 2024. A visualization of the conserved motifs in *FADs* was performed using the TBtools (v2.080) [42] Gene structures were analyzed using the Gene Structure Display Server (GSDS, http://gsds.gao-lab.org/), accessd on 15 January 2024. ExPASy Protparam (https://web.expasy.org/protparam/) was employed to analyze the physicochemical properties of the identified proteins, accessd on 20 January 2024. Additionally, WOLF PSORT (https://wolfpsort.hgcip) was utilized to predict the subcellular localization of the *FAD* family members, accessd on 20 January 2024. Upstream sequences of the FAD-coding sequences (2 kb) were extracted from genome using TBtools [42] and subjected to a cis-acting elements prediction analysis using PlantCARE [27] (http://bioinformatics.psb.ugent.be/webtools/plantcare/html/, accessd on 20 January 2024). To better illustrate the enrichment of motifs in the promoters of *FAD* genes, motifs with a total count of less than 10 across all genes were removed.

### 4.3. Chromosomal Location and Duplication Events of FAD Genes

We performed a sequence alignment of all FAD protein sequences using the local BlastP program with an e-value threshold of 1 × 10^−10^ and considering the top 5 alignments. The resulting BlastP outputs, combined with genomic annotation files, were then used as the input for generating collinearity and tandem files through MCScanX [43]. The collinearity analysis was meticulously conducted and visualized using MCScanX (https://github.com/wyp1125/MCScanx) and TBtools (v2.080) [42,43]. We explored the interrelationship between oil tea and tea using JCVI (v1.3.5) (https://github.com/tanghaibao/jcvi) and visualized the collinearity between the four species.

### 4.4. Comparative Expression Analysis of the FAD Genes in Seeds of Tea and Oil Tea

The samples we utilized for our transcriptome analysis were taken from mature seeds. Seed materials from *C. lanceoleosa*, *C. oleifera*, and *C. sinensis* var. *sinensis TGY* were collected and stored at −80 °C for the subsequent analysis. The Hieff NGS^®^ MaxUpTM II Dual-mode mRNA Library Prep Kit for Illumina^®^ (Yeasen, Shanghai, China) was employed to prepare mRNA libraries for sequencing on Illumina platforms. After the library preparation and pooling of different samples, they underwent Illumina sequencing. All experiments were biologically replicated three times. The quality assessment and filtering of original sequencing data were performed using Fastp (v0.23.2) software [44]. The sequenced reads were aligned to the reference genome of *C. lanceoleosa* using Hisat2 [45]. The gene expression quantification of the aligned reads was conducted using StringTie [46]. Expression heatmaps were generated using the R package TOmicsVis [47] to visualize and analyze gene expression patterns.

### 4.5. Quantitative RT-PCR (qRT-PCR) in Seeds of Tea and Oil Tea

A RNAprep Pure Plant Plus Kit was used to extract RNA from samples. cDNA was synthesized with an M-MLV 4 First-Strand cDNA Synthesis Kit and qRT-PCR was conducted using Green qPCR MasterMix. Relative transcript abundance was calculated using the comparative 2^−ΔΔC_T_^ method. *EF1α* was selected as the internal control for qRT-PCR and the primers utilized in the study are detailed in supplementary Table S1.

### 4.6. Protein Clustering and Analyzing

For protein structure prediction and visualization, we relied on the FastAF2 tool (https://cloud.zelixir.com/fastaf2/#/fast-af2, accessed on 21 November 2023). We specifically focused on obtaining high-confidence protein structures by filtering them with an average per-residue confidence metric called predicted local distance difference test (pLDDT) greater than 80. We utilized the TMscoring algorithm to assess the structural similarity between pairs of protein structures. The TM-score provided a quantitative measure of similarity, while RMSD quantified the deviation between corresponding atoms. For each protein structure pair, we calculated both TM-score and RMSD values. By assigning weights to TM-score and normalized RMSD, we derived a combined score that accurately represented the overall structural similarity.

To cluster the protein structures, we constructed a similarity matrix based on the combined scores. The Unweighted Pair Group Method with Arithmetic Mean (UPGMA) algorithm was then applied to the similarity matrix, generating a hierarchical clustering tree. This clustering tree offered an intuitive representation of the relationships between the protein structures. To visualize the clustering tree, we used the iTOL (https://itol.embl.de/) platform. In addition, the FAD protein–protein interaction network was analyzed using the STRING database (https://cn.string-db.org/) to identify hub genes. After establishing the protein–protein interaction (PPI) relationship, a network diagram was created using Cytoscape [48] (v3.10.1) software. Hub genes were identified within the network based on degree centrality.

## Data Availability

The protein sequences for *C. oleifera* were stored in a GitHub repository, which is publicly accessible at https://github.com/Hengfu-Yin/CON_genome_data, accessed on 19 July 2023. Protein sequences for *C. sinensis* and *C. chekiangoleosa* were deposited in the Genome Warehouse (GWH) at the National Genomics Data Center, Beijing Institute of Genomics (Chinese Academy of Sciences), and can be accessed with the accession numbers GWHASIV00000000 and GWHBGBN00000000, respectively. Reference protein sequences for *O. sativa* and *A. thaliana* were downloaded from the Phytozome database (available via https://phytozome-next.jgi.doe.gov/), accessed on 19 July 2023. The transcriptome data of *C. chekiangoleosa* seeds can be accessed from the Sequence Read Archive (SRA) using the accession number PRJNA753883.

## Supplementary Materials

The following supporting information can be downloaded at: https://www.mdpi.com/article/10.3390/plants13111444/s1, Figure S1: Orthologous cluster analysis of eight plant species, including *C. oleifera*, *C. chekiangoleosa*, *C. lanceoleosa*, *A. thaliana*, *O. sativa*, *C. sinensis*, *V. vinifera*, and *A. chinensis*. Figure S2: Gene structure diagram illustrating the features of *FAD* genes. Coding sequences (CDS) are represented in green, while untranslated regions (UTR) are depicted in yellow. Figure S3: Distribution of cis-acting elements in the promoters of FAB2(SAD) subfamily from tea and oil tea. Figure S4: Comprehensive synteny analysis of *C. sinensis* (Cs), *C. oleifera* (Co), *C. chekiangoleosa* (Cc), and *C. lanceoleosa* (Cl). Figure S5: Predicted three-dimensional structures of FAD proteins, except the FAB2(SAD) proteins from tea and oil tea. Figure S6: Predicted three-dimensional structures of FAB2(SAD) proteins from tea and oil tea. Table S1: Details of primers used in this study. Table S2: Characteristics of *FAD* gene family in tea and oil tea.
