# Supplementary material for: Comparative Genome-Wide Identification of the Fatty Acid Desaturase Gene Family in Tea and Oil Tea
Source: Plants (Basel). 2024 May 23;13(11):1444. doi: 10.3390/plants13111444 (PMC11174766; doi:10.3390/plants13111444)
Supplement: Supplementary file 1 [file plants-13-01444-s001.zip › Supplementary-Figure S6.pdf]

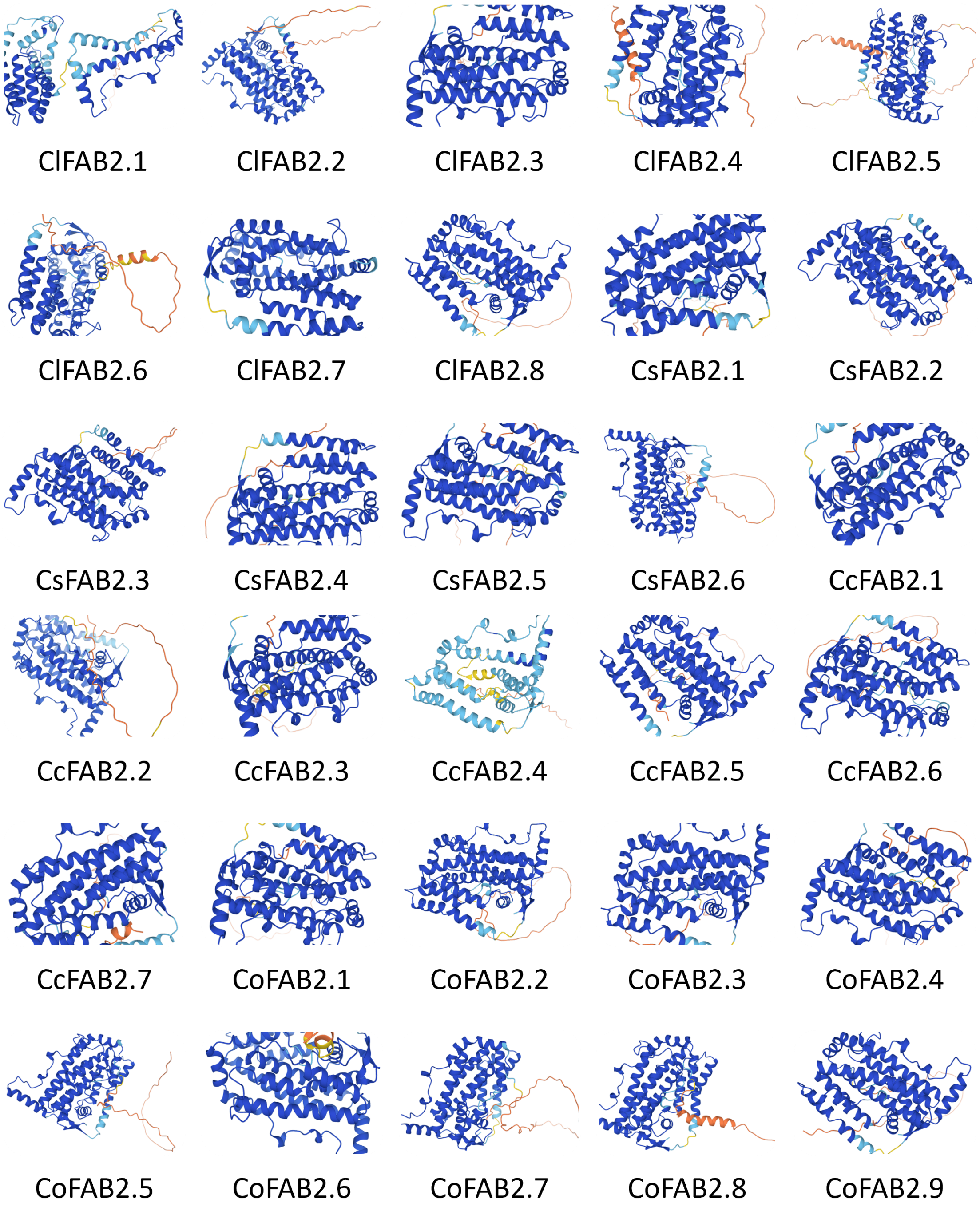
ClFAB2.1
ClFAB2.2
ClFAB2.3
ClFAB2.4
ClFAB2.5
ClFAB2.6
ClFAB2.7
ClFAB2.8
CsFAB2.1
CsFAB2.2
CsFAB2.3
CsFAB2.4
CsFAB2.5
CsFAB2.6
CcFAB2.1
CcFAB2.2
CcFAB2.3
CcFAB2.4
CcFAB2.5
CcFAB2.6
CcFAB2.7
CoFAB2.1
CoFAB2.2
CoFAB2.3
CoFAB2.4
CoFAB2.5
CoFAB2.6
CoFAB2.7
CoFAB2.8
CoFAB2.9
