# Supplementary figures and images for: Comparative Genome-Wide Identification of the Fatty Acid Desaturase Gene Family in Tea and Oil Tea
Source: Plants (Basel). 2024 May 23;13(11):1444. doi: 10.3390/plants13111444 (PMC11174766; doi:10.3390/plants13111444)

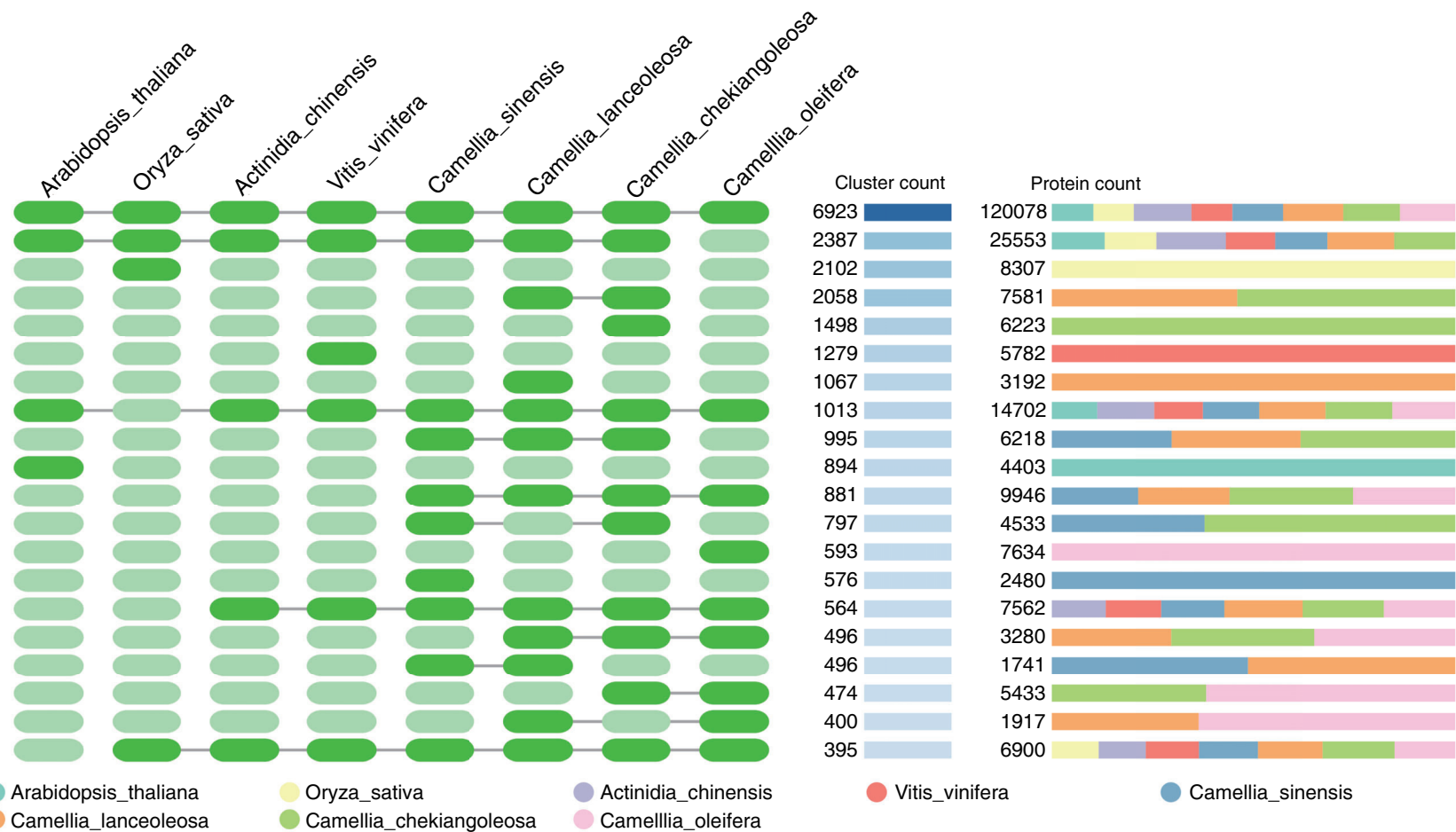

Supplement: Supplementary file 1 [file plants-13-01444-s001.zip › Supplementary-Figure S1.pdf]

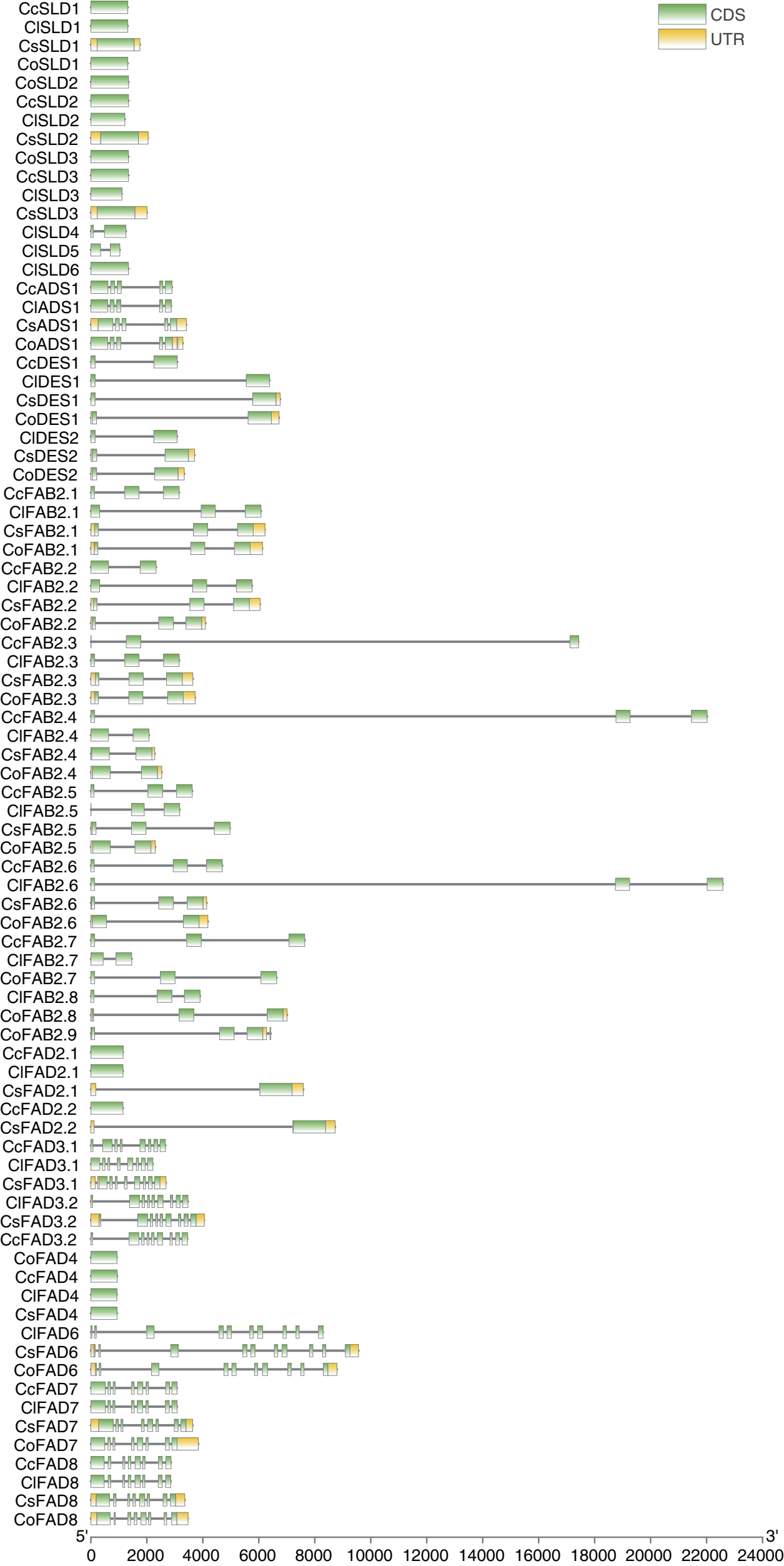

Supplement: Supplementary file 1 [file plants-13-01444-s001.zip › Supplementary-Figure S2.pdf]

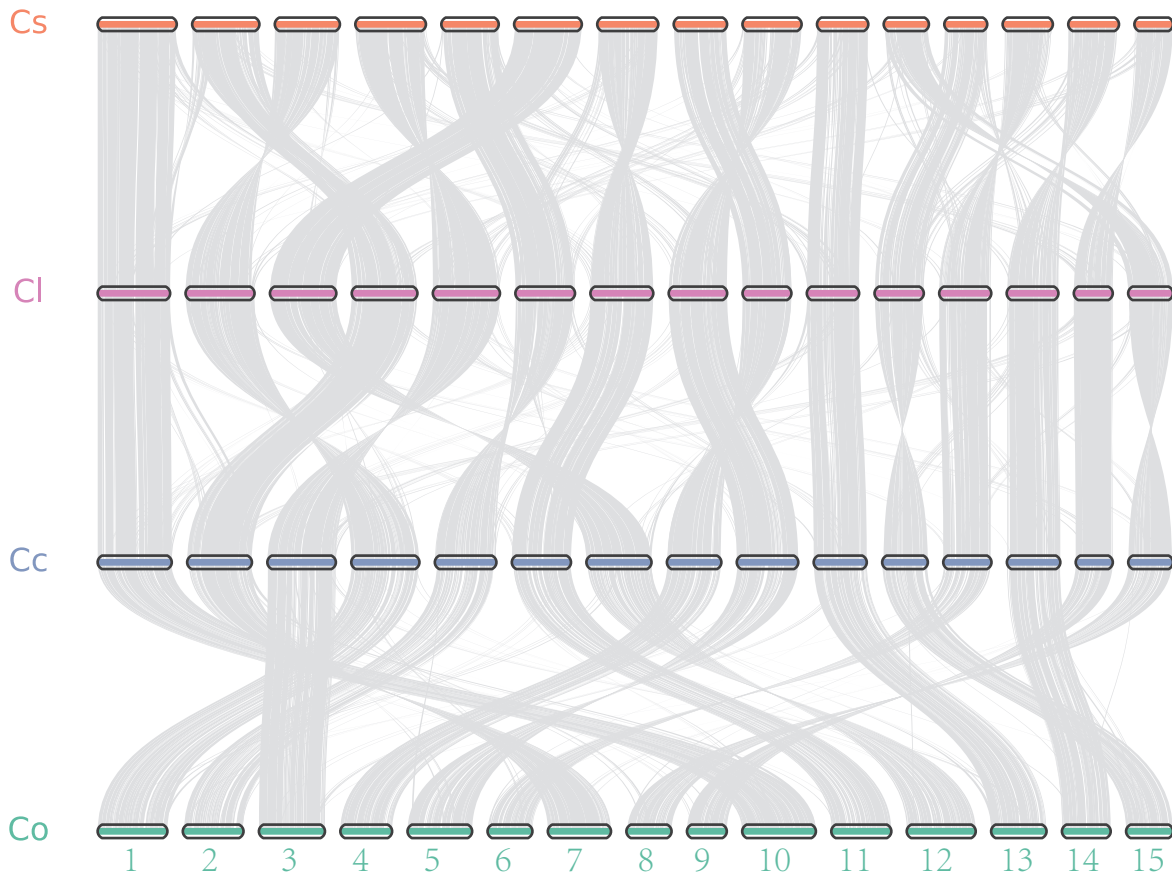

Supplement: Supplementary file 1 [file plants-13-01444-s001.zip › Supplementary-Figure S4.pdf]

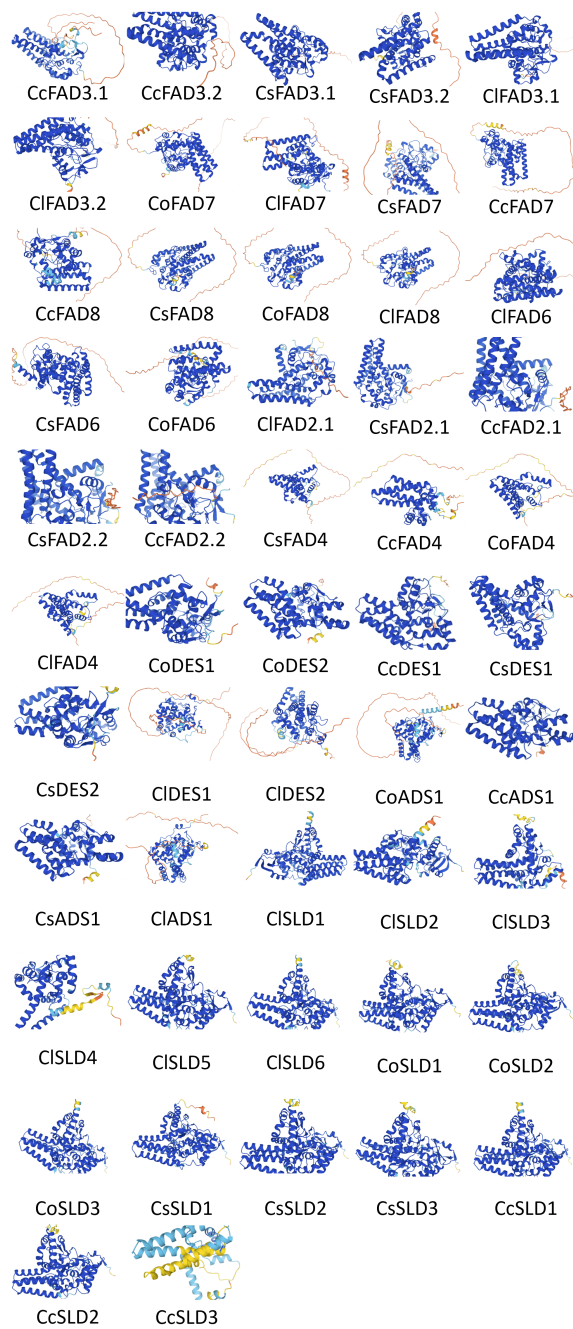

Supplement: Supplementary file 1 [file plants-13-01444-s001.zip › Supplementary-Figure S5.pdf]
