# Supplementary material for: Comparative Genome-Wide Identification of the Fatty Acid Desaturase Gene Family in Tea and Oil Tea
Source: Plants (Basel). 2024 May 23;13(11):1444. doi: 10.3390/plants13111444 (PMC11174766; doi:10.3390/plants13111444)
Supplement: Supplementary file 1 [file Supplementary-Figure S3.pdf › Supplementary-Figure S3.pdf]

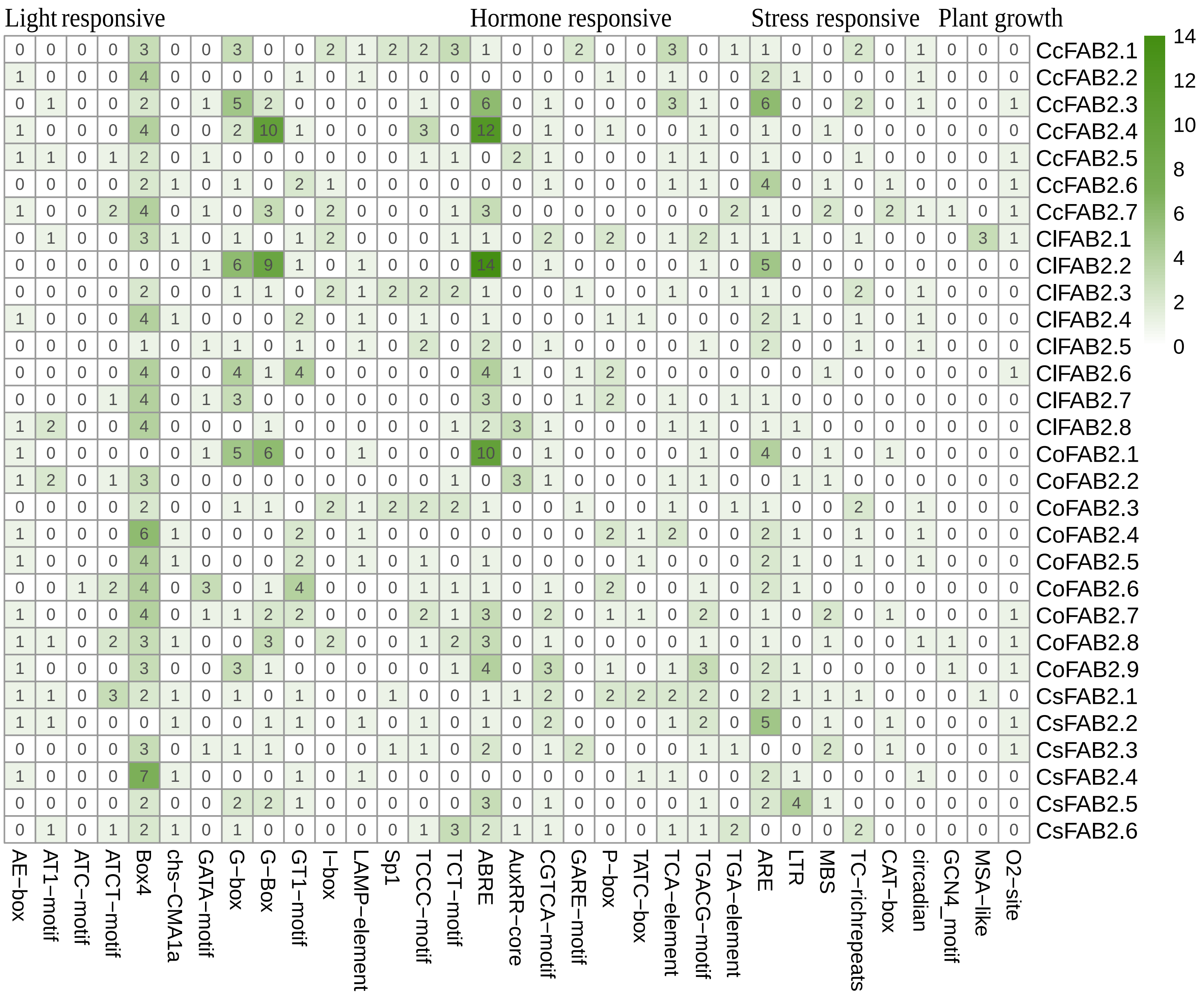

Light responsive
Hormone responsive
Stress responsive
Plant growth
AE−box
AT1−motif
ATC−motif
ATCT−motif
Box4
chs−CMA1a
GATA−motif
G−box
G−Box
GT1−motif
I−box
LAMP−element
Sp1
TCCC−motif
TCT−motif
ABRE
AuxRR−core
CGTCA−motif
GARE−motif
P−box
TATC−box
TCA−element
TGACG−motif
TGA−element
ARE
LTR
MBS
TC−richrepeats
CAT−box
circadian
GCN4_motif
MSA−like
O2−site
CcFAB2.1 0 0 0 0 3 0 0 3 0 0 2 1 2 2 3 1 0 0 2 0 0 3 0 1 1 0 0 2 0 1 0 0 0
CcFAB2.2 1 0 0 0 4 0 0 0 0 1 0 1 0 0 0 0 0 0 0 1 0 1 0 0 2 1 0 0 0 1 0 0 0
CcFAB2.3 0 1 0 0 2 0 1 5 2 0 0 0 0 1 0 6 0 1 0 0 0 3 1 0 6 0 0 2 0 1 0 0 1
CcFAB2.4 1 0 0 0 4 0 0 2 10 1 0 0 0 3 0 12 0 1 0 1 0 0 1 0 1 0 1 0 0 0 0 0 0
CcFAB2.5 1 1 0 1 2 0 1 0 0 0 0 0 0 1 1 0 2 1 0 0 0 1 1 0 1 0 0 1 0 0 0 0 1
CcFAB2.6 0 0 0 0 2 1 0 1 0 2 1 0 0 0 0 0 0 1 0 0 0 1 1 0 4 0 1 0 1 0 0 0 1
CcFAB2.7 1 0 0 2 4 0 1 0 3 0 2 0 0 0 1 3 0 0 0 0 0 0 0 2 1 0 2 0 2 1 1 0 1
ClFAB2.1 0 1 0 0 3 1 0 1 0 1 2 0 0 0 1 1 0 2 0 2 0 1 2 1 1 1 0 1 0 0 0 3 1
ClFAB2.2 0 0 0 0 0 0 1 6 9 1 0 1 0 0 0 14 0 1 0 0 0 0 1 0 5 0 0 0 0 0 0 0 0
ClFAB2.3 0 0 0 0 2 0 0 1 1 0 2 1 2 2 2 1 0 0 1 0 0 1 0 1 1 0 0 2 0 1 0 0 0
ClFAB2.4 1 0 0 0 4 1 0 0 0 2 0 1 0 1 0 1 0 0 0 1 1 0 0 0 2 1 0 1 0 1 0 0 0
ClFAB2.5 0 0 0 0 1 0 1 1 0 1 0 1 0 2 0 2 0 1 0 0 0 0 1 0 2 0 0 1 0 1 0 0 0
ClFAB2.6 0 0 0 0 4 0 0 4 1 4 0 0 0 0 0 4 1 0 1 2 0 0 0 0 0 0 1 0 0 0 0 0 1
ClFAB2.7 0 0 0 1 4 0 1 3 0 0 0 0 0 0 0 3 0 0 1 2 0 1 0 1 1 0 0 0 0 0 0 0 0
ClFAB2.8 1 2 0 0 4 0 0 0 1 0 0 0 0 0 1 2 3 1 0 0 0 1 1 0 1 1 0 0 0 0 0 0 0
CoFAB2.1 1 0 0 0 0 0 1 5 6 0 0 1 0 0 0 10 0 1 0 0 0 0 1 0 4 0 1 0 1 0 0 0 0
CoFAB2.2 1 2 0 1 3 0 0 0 0 0 0 0 0 0 1 0 3 1 0 0 0 1 1 0 0 1 1 0 0 0 0 0 0
CoFAB2.3 0 0 0 0 2 0 0 1 1 0 2 1 2 2 2 1 0 0 1 0 0 1 0 1 1 0 0 2 0 1 0 0 0
CoFAB2.4 1 0 0 0 6 1 0 0 0 2 0 1 0 0 0 0 0 0 0 2 1 2 0 0 2 1 0 1 0 1 0 0 0
CoFAB2.5 1 0 0 0 4 1 0 0 0 2 0 1 0 1 0 1 0 0 0 0 1 0 0 0 2 1 0 1 0 1 0 0 0
CoFAB2.6 0 0 1 2 4 0 3 0 1 4 0 0 0 1 1 1 0 1 0 2 0 0 1 0 2 1 0 0 0 0 0 0 0
CoFAB2.7 1 0 0 0 4 0 1 1 2 2 0 0 0 2 1 3 0 2 0 1 1 0 2 0 1 0 2 0 1 0 0 0 1
CoFAB2.8 1 1 0 2 3 1 0 0 3 0 2 0 0 1 2 3 0 1 0 0 0 0 1 0 1 0 1 0 0 1 1 0 1
CoFAB2.9 1 0 0 0 3 0 0 3 1 0 0 0 0 0 1 4 0 3 0 1 0 1 3 0 2 1 0 0 0 0 1 0 1
CsFAB2.1 1 1 0 3 2 1 0 1 0 1 0 0 1 0 0 1 1 2 0 2 2 2 2 0 2 1 1 1 0 0 0 1 0
CsFAB2.2 1 1 0 0 0 1 0 0 1 1 0 1 0 1 0 1 0 2 0 0 0 1 2 0 5 0 1 0 1 0 0 0 1
CsFAB2.3 0 0 0 0 3 0 1 1 1 0 0 0 1 1 0 2 0 1 2 0 0 0 1 1 0 0 2 0 1 0 0 0 1
CsFAB2.4 1 0 0 0 7 1 0 0 0 1 0 1 0 0 0 0 0 0 0 0 1 1 0 0 2 1 0 0 0 1 0 0 0
CsFAB2.5 0 0 0 0 2 0 0 2 2 1 0 0 0 0 0 3 0 1 0 0 0 0 1 0 2 4 1 0 0 0 0 0 0
CsFAB2.6 0 1 0 1 2 1 0 1 0 0 0 0 0 1 3 2 1 1 0 0 0 1 1 2 0 0 0 2 0 0 0 0 0
14
12
10
8
6
4
2
0
